# FIP1L1‐PDGFRA fusion gene in T‐lymphoblastic lymphoma: A case report

**DOI:** 10.1002/cnr2.1769

**Published:** 2022-12-14

**Authors:** Salhah Ali, Yasmine Al‐Qattan, Walaa Awny, Abdulaziz Hamadah, Karen Pinto, Salem AlShemmari

**Affiliations:** ^1^ Department of Hematology Mubarak Al‐Kabeer Hospital Jabriya Kuwait; ^2^ Department of Hematopathology Kuwait Cancer Control Center Kuwait Kuwait; ^3^ Department of Hematology Kuwait Cancer Control Center Kuwait Kuwait; ^4^ Department of Pathology Kuwait Cancer Center Kuwait Kuwait; ^5^ Department of Medicine, Faculty of Medicine Kuwait University Kuwait Kuwait

**Keywords:** FIP1L1‐PDGFRA, T lymphoblastic lymphoma, TLL, eosinophilia

## Abstract

**Background:**

T‐lymphoblastic lymphoma (T‐LBL) is an aggressive malignancy of T‐lymphoid precursors, rarely co‐occurring with myeloid/lymphoid neoplasms with eosinophilia (M/LNs‐Eo), with consequent rearrangement of tyrosine kinase (TK)‐related genes. The FIP1L1‐PDGFRA fusion gene is the most frequent molecular abnormality seen in eosinophilia‐associated myeloproliferative disorders, but is also present in acute myeloid leukemia (AML), T‐lymphoblastic leukemia/lymphoma (TLL), or both simultaneously. T‐LBL mainly affects children and young adults, involving lymph node, bone marrow, and thymus. It represents about 85% of all immature lymphoblastic lymphomas, whereas immature B‐cell lymphomas comprise approximately 15% of all cases of LBL.

**Case:**

In this case report, we present an example of T cell lymphoblastic lymphoma with coexistent eosinophelia, treated successfully with a tyrosine‐kinase inhibitor (TKI).

**Conclusion:**

FIP1L1‐PDGFRA‐positive T‐LBL and myeloproliferative disorders have excellent response to low‐dose treatment with (TKI) imatinib. Most patients achieve rapid and complete hematologic and molecular remission within weeks.

## 1 INTRODUCTION

T‐lymphoblastic lymphoma (T‐LBL) is an aggressive malignancy of T‐lymphoid precursors, which can rarely co‐occur with Myeloid/Lymphoid Neoplasms with Eosinophilia (M/LNs‐Eo).^1^ Acquired genetic and epigenetic aberrations affect the early thymocyte subset leading to the formation of T‐LBL and T‐ALL malignancies. T‐ALL and T‐LBL are commonly found amongst children and young adults, manifesting in 15% of the ALL cases, whereas T‐LBL affects 20% of the non‐Hodgkin lymphomas (NHLs).^2^ The World Health Organization (WHO) designated T‐ALL and T‐LBL as T‐lymphoblastic leukemia/lymphoma in the updated Revised WHO classification, without further specification. Another classification clearly defined as a category of three related disease entities by the revised WHO is termed myeloid and lymphoid neoplasms with eosinophilia and abnormalities of PDGFRA, PDGFRB, and FGFR. These fusion genes encode an aberrant tyrosine kinase, which usually manifest with eosinophilia in the peripheral blood.^3^ Here we present a case of a T‐LBL with eosinophilia found to have FIP1L1‐PDGFRA rearrangement.

## 2 CASE PRESENTATION

A 53‐year‐old previously healthy male was referred to Kuwait Cancer Control Centre (KCCC) with a 1‐year history of multiple painless lumps. He tested positive for SARS‐COV2 in March 2020, with a non‐complicated course requiring home quarantine only. After that, he started to develop numerous painless lumps; the first one appeared in the right submandibular area, which he attributed to COVID‐19 infection. Later he developed another painless swelling in the right inguinal area, after which he sought medical attention. No other symptoms of note, no weight loss, night sweats, or fever.

On examination, he was hemodynamically stable, conscious, oriented, afebrile.

Chest and cardiac examinations were unremarkable. Abdominal examination revealed a splenomegaly, with the spleen felt two centimeters below the left costal margin. Upon examining the lymph nodes, a right submandibular 2 × 2 cm's painless mass, without overlying skin changes. Another bigger swelling was felt in the right inguinal area, measuring 3 × 3 cm's, fixed, rubbery, and no overlying skin changes.

**FIGURE 1:**
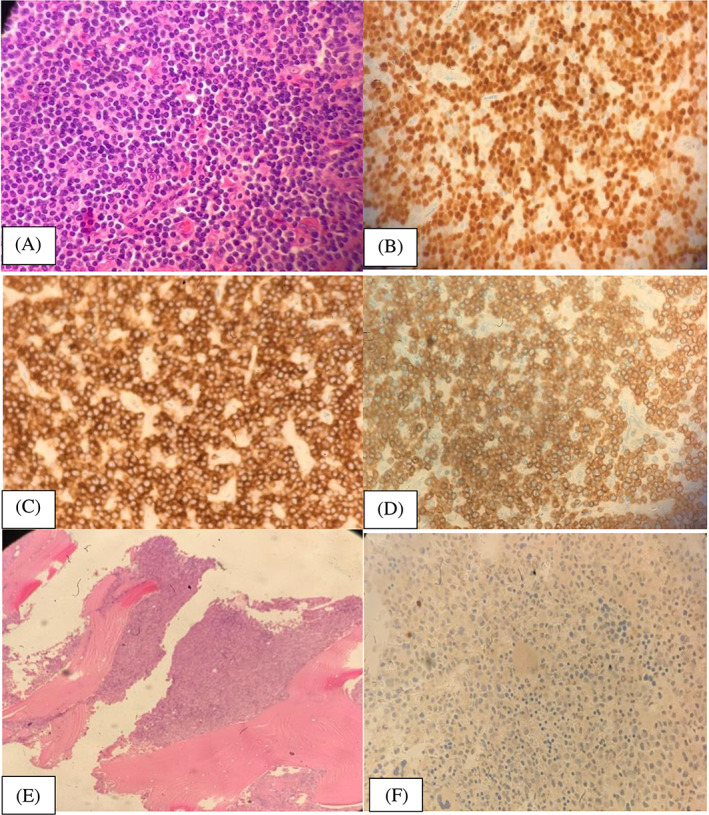
(A) Lymph node histopathology, (B) lymph node immunohistochemistry for TDT, (C) is lymph node immunohistochemistry staining for CD7, (D) is lymph node immunohistochemistry for CD3, (E) shows a hypercellular marrow pre‐treatment and (F) is a negative TdT staining at diagnosis

**FIGURE 2:**
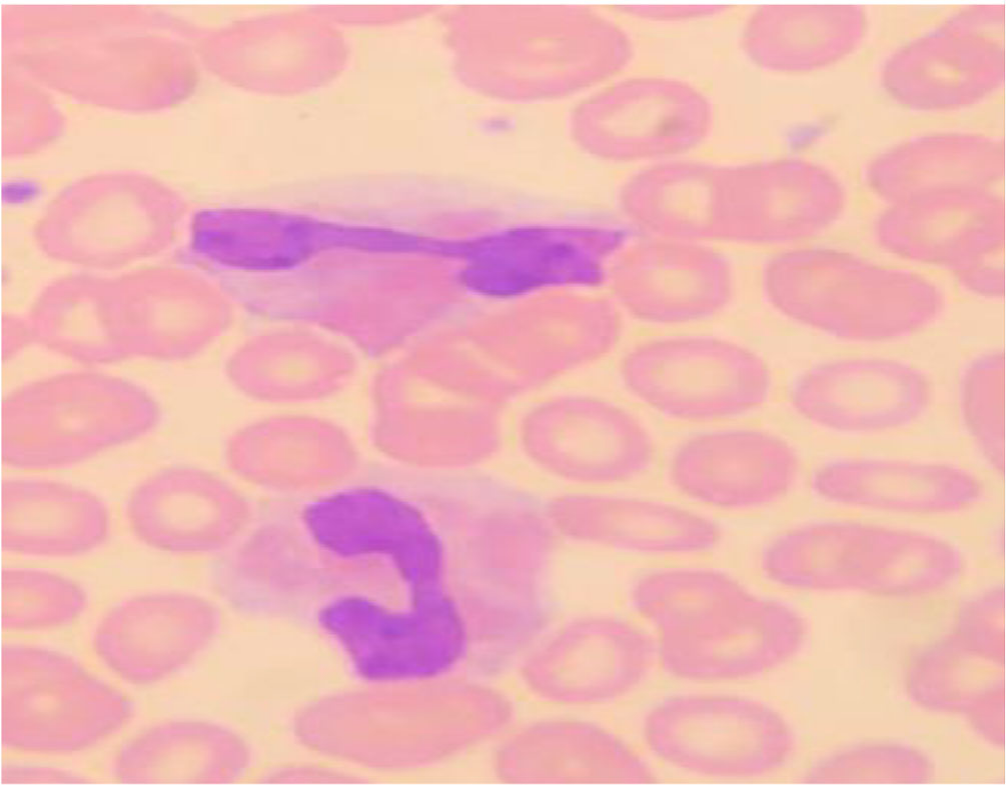
Demonstrates peripheral eosinophilia with dysplasia and hypogranulation

**FIGURE 3:**
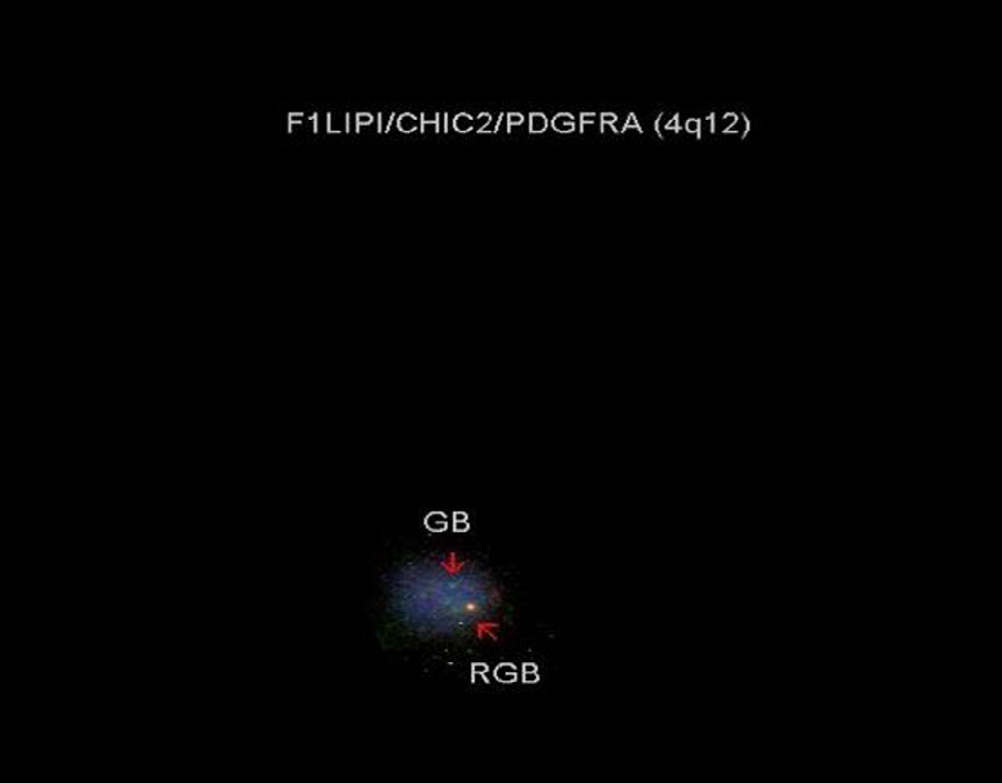
Shows interstitial deletion of CHIC2 gene found on FISH using FIP1L1/CHIC2/PDGFRA probe

**FIGURE 4:**
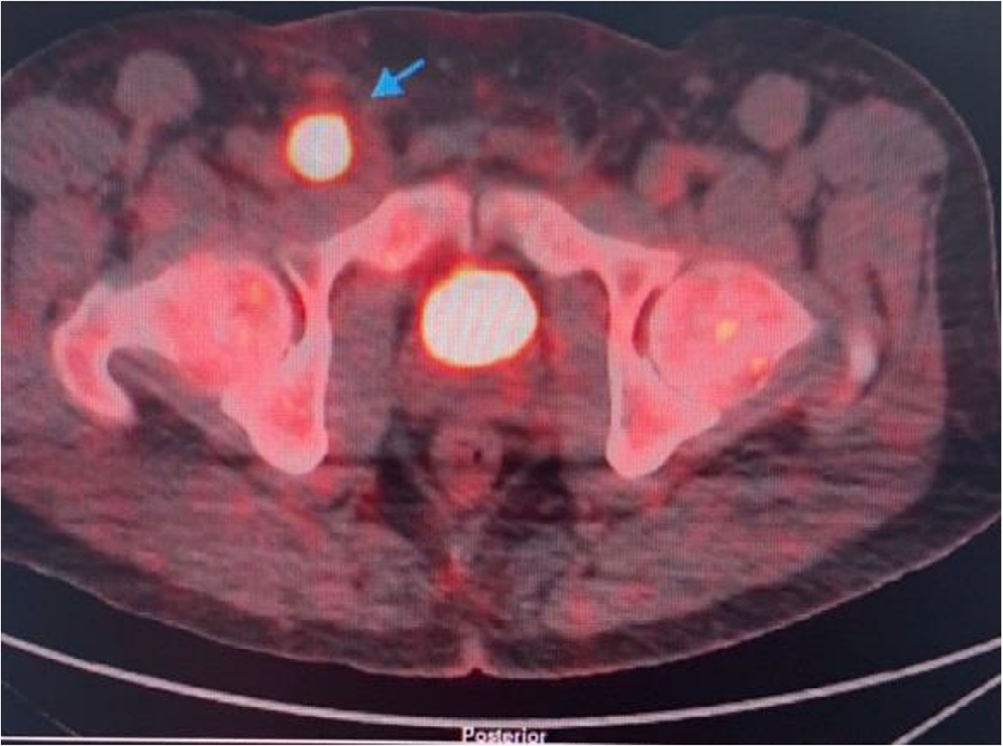
A transverse view of FDG PET showing a hypermetabolic right external iliac and inguinal lymph nodes with SUV max 15.8

Patient lab. Investigations:

- Full blood count showed absolute eosinophilia as shown in Table 1.
- Biochemical parameters such as Creatinine, Alkaline phosphatase, Total bilirubin, Total protein and electrolytes were in normal range. However, Uric acid was found to be elevated at 585 μmol/L (normal range 208–428 μmol/L).
- Virology screen negative for Hepatitis B, C, and HIV.
- Excisional biopsy from the Rt inguinal LN: T‐lymphoblastic Lymphoma (TLL), positive for CD3, CD2, CD7, TDT as shown in Figure 1. Negative for Cyclin D1, CD20, PAX5, and Bcl6.
- Blood Film: WBC showed absolute eosinophilias shown in Figure 2, with eosinophils showing dysplasia and hypogranulation. Red blood cells are normocytic normochromic cells. His platelet count was adequate.
- Ultrasound (US) of inguinal lump showed a suspicious hypoechoic lesion possibly nodal for fine needle aspiration cytology, which in turn showed necrotic material.
- PAN CT (Neck, chest, abdomen and pelvis):
  - Multiple variable‐sized enlarged cervical lymph nodes are seen bilaterally; some show globular appearance with attenuated hilum, largest is seen at right Ib group measuring about 17 × 23 × 18 mm (suspicious).
  - Bilateral posterior basal faint ground‐glass opacities (previous Covid infection).
  - Well‐defined globular shape large lymph node is seen at the right inguinal region, measuring about 2.4 × 3 × 3.6 cm at maximum diameters. It shows no cystic breakdown or abscess formation with no intra‐abdominal extension. Preserved surrounded fat plane as well. Other few small inguinal lymph nodes are noted.
  - The liver is mildly enlarged, has homogenous attenuation, no focal or enhancing lesions; no intra or extra hepatic biliary dilation.
  - Enlarged spleen with a span measuring about 15 cm. No focal lesions.
- Bone marrow biopsy and aspirate:
  - Aspirate: hypercellular marrow, with an eosinophil percentage of approximately 2%. No morphological evidence of bone marrow infiltration by lymphoma on aspiration smear.
  - Biopsy: No infiltration by lymphoma cells.
  - Myeloid/lymphoid neoplasm with PDGFRA rearrangement.
  - Trephine biopsy shows scattered small CD20/CD79a‐ve+ B‐lymphocytes and CD3/CD5‐VE+ T‐Lymphocytes. No lymphoma infiltration.
- Marrow aspirate cytogenetics analysis: 46, XY (normal karyotype). Fluorescence In Situ Hybridization (FISH) from peripheral blood: FIP1L1/CHIC2/PDGFRA: 70% interstitial deletion detected (Figure 3).
- FDG‐PET done: Hypermetabolic lymphadenopathy below and above the diaphragm.
  - Diffuse hypermetabolic bone marrow in the axial and proximal appendicular skeleton (Figure 4).

**TABLE 1:** Blood investigations of the patient—complete blood count (CBC)

	Result	Normal range
White blood cells (WBC)	7.3 × 10^9^/L	3.8–11/L
Hemoglobin	154 g/L	140–180 g/L
Hematocrit	0.497	0.36–0.46
Mean corpuscular volume (MCV)	82.4 fl	80–94 fl
Platelet count	147/L (L)	150–450/L
Neutrophils #	2.17/L	1.7–7.1/L
Lymphocyte #	2.04/L	1–3.5/L
Monocytes #	0.68/ L	0.3–0.8/L
Eosinophils #	2.24/L (H)	0–0.5/L

## 3 TREATMENT COURSE

**FIGURE 5:**
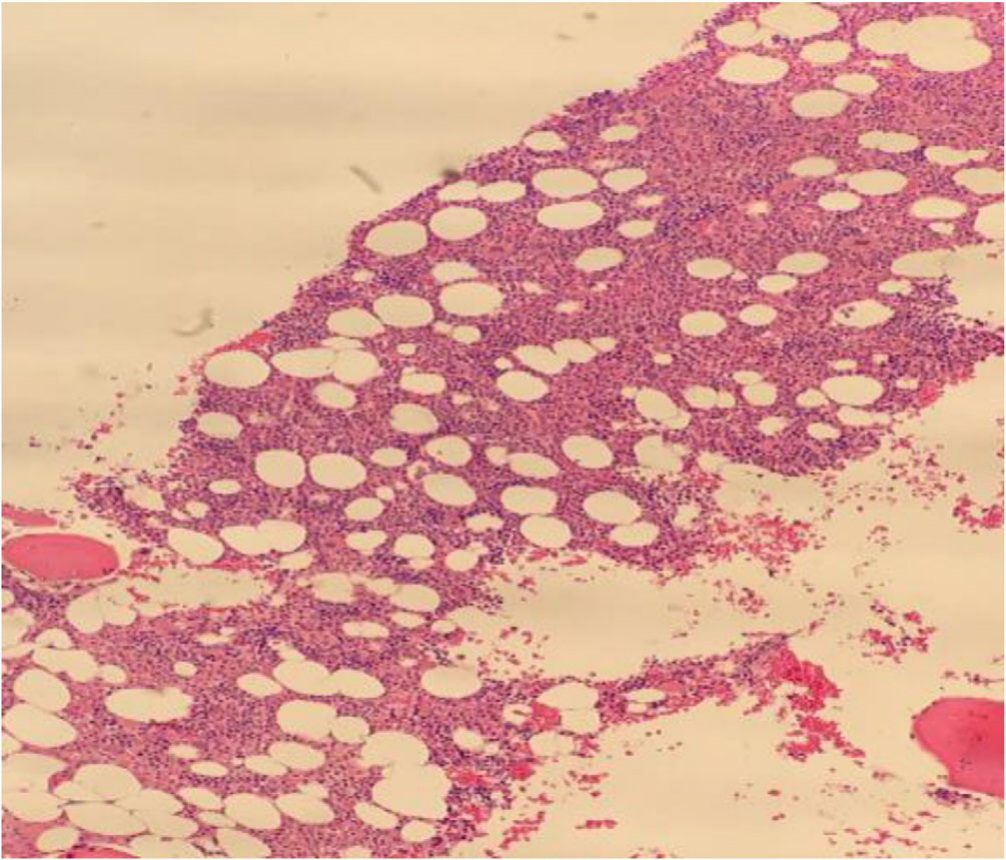
Is a normocellular marrow 1 month post treatment with TKIs

- The patient was diagnosed as a case of T‐lymphoblastic lymphoma with FIP1L1/PDGFRA rearrangement and was started on a tyrosine kinase inhibitor (TKI) imatinib 100 mg once daily. His symptoms improved with treatment. Bone marrow aspirate and biopsy were repeated after 1 month and showed complete resolution of eosinophilia as shown in Figure 5. Interim FDG PET showed complete resolution of the hypermetabolic lesions. Immunophenotyping showed only mature T‐lymphocytes with normal CD4/CD8 ratio and no expression of B‐lymphocyte markers. FISH analysis from the bone marrow aspirate was negative for the FIP1L1‐PDGFRA fusion gene, which is considered a complete response to TKI.

## 4 DISCUSSION

FIP1‐like‐1 (FIP1L1)‐Platelet‐Derived Growth Factor Receptor Alpha (PDGFRA) fusion gene is a recurrent molecular abnormality, most frequently seen in eosinophilia‐associated myeloproliferative disorders (Eos‐MPD). However, it can also present in acute myeloid leukemia (AML), T‐lymphoblastic leukemia/lymphoma (TLL) or both simultaneously. Infact, it is believed to be the most common fusion gene seen in myeloproliferative neoplasms associated with PDGRFA rearrangement. The fusion gene can be detected by reverse transcriptase–polymerase chain reaction (RT‐PCR), or nested RT‐PCR more often, or by FISH analysis using a probe for CHIC2 gene deletion, or using a break‐apart probe that encompasses FIP1L1 and PDGFRA.^4^

Other molecular variants of FIP1L1‐PDGFRA‐associated MPD have been identified, where other fusion genes have incorporated as part of PDGFRA. Examples include: KIF5B‐PDGFRA,^5^ STRN‐PDGFRA, ETV6‐PDGFRA,^6^ CDK5RAP2‐PDGFRA,^7^ and BCR‐PDGFRA.^8^

This fusion gene is a result of a cryptic deletion at 4q12, which is usually not visible by standard cytogenetic banding methods, giving us an apparently normal karyotype. The fusion gene produces a fusion protein with an increased tyrosine kinase activity. This subsequently led to the fact that excellent response was found to imatinib. It is believed to be 100‐fold more sensitive to imatinib than to BCR‐ABL.^9^ Our case exhibited a 70% interstitial CHIC2 gene deletion using FISH analysis.

T‐LBL is a rare and aggressive form of precursor T‐cell non‐Hodgkin's Lymphomas (T‐NHLs) affecting mainly children and young adults and involving lymph nodes, BM and thymus.^1^ T‐LBL represents around 85% of all immature lymphoblastic lymphomas, whereas immature B‐cell.

Lymphomas make up approximately 15% of all LBL cases.^10^ T lymphoblastic lymphoma has a high association with leukemia and low 5‐year survival.^11^ Various treatment modalities have been used in this disease including intensive chemotherapy and hematopoietic stem cell transplantation, but the mortality rate remains high.

Rarely, as in our case, T‐LBL can develop in the context of Myeloid and Lymphoid Neoplasms with Eosinophilia and rearrangement of tyrosine‐kinase (TK) genes. Eosinophilia is commonly found in T‐NHLs and it is believed to be reactive in most cases, due to the overproduction of cytokines such as IL3, IL5, and GM‐CSF.^4^ Clonal eosinophilia in association with T‐NHL is generally very rare. Therefore, accurate diagnosis and classification of T‐LBL has an important significance as it implicates management planning and decisions. Recently, FIP1L1‐PDGFRA‐positive myeloproliferative neoplasms appeared to have a very good response to low (50–100 mg per day) or intermittent (once daily to once weekly) doses of imatinib (FIP1L1‐PDGFRA in eosinophilic disorders). Most of the patients achieve rapid complete hematologic and complete molecular remission within weeks. Imatinib, a tyrosine kinase inhibitor (TKI), is proven to be effective in treating clonal eosinophilia with good reported response rates, as it can selectively inhibit BCR‐ABL, C‐KIT, and platelet‐derived growth factor receptors A and B (PDGFRA and PDGFRB).^11^

Our case is an example of T cell lymphoblastic lymphoma with coexistent eosinophilia. He was treated with low dose imatinib (100 mg once daily) as he had FIP1L1‐PDGFRA rearrangement. He showed an excellent response to TKI's and is currently under regular follow‐up.

## 5 CONCLUSION

As aforementioned, our case report shows a rare presentation of TLL with eosinophilia found to have FIP1L1‐PDGFRA rearrangement. Our patient was treated with a chemotherapy‐free regimen using Tyrosine Kinase Inhibitors with low dose imatinib and showed a great response with complete resolution of his lesions on PET scan along with normal bone marrow on follow up assessment. Our patient's excellent response to low‐dose TKI is an example of what the future of haemato‐oncology holds for such patients who are diagnosed with illnesses that were once—and still are—viewed as sinister. Reporting of such cases and their optimal outcomes may pave the way for other haemato‐oncologists who are also looking to achieve good outcomes using treatments other than the traditional chemotherapy route. We believe that TKIs are just the tip of the iceberg when it comes to treating the different haemato‐oncological diseases as more and more novel treatments are emerging daily, and the reporting of patients' outcomes when receiving these treatments is how optimal regimens are implemented.

## AUTHOR CONTRIBUTIONS

**Salhah Ali:** Conceptualization (equal); writing – original draft (equal); writing – review and editing (equal). **Yasmine Al‐Qattan:** Data curation (equal); resources (equal); writing – original draft (equal). **Walaa Awny:** Investigation (equal); resources (equal); supervision (equal). **Abdulaziz Hamadah, Karen Pinto & Salem AlShemmari:** Investigation (equal); resources (equal); writing – original draft (equal).

## CONFLICT OF INTEREST

The authors of this paper have no conflicts of interest, including specific financial interests, relationships, and/or affiliations relevant to the subject matter or materials included.

## ETHICS STATEMENT

No institutional review board approval was required as retrospective review of patient case for publication is not considered human subjects' research. Therefore, no patient consent was required.

## INFORMED CONSENT

The patient participated voluntarily and personally provided his informed consent for the publishing of their medical information.

## Data Availability

Data sharing is not applicable to this article as no new data were created or analyzed in this study.
